# Foehn: An open-hardware asynchronous scheduling hub for high-throughput liquid-handling workflow

**DOI:** 10.1016/j.ohx.2026.e00748

**Published:** 2026-02-03

**Authors:** Yueyang Gao, Jacob Danks, Simon Dawes, Maximilian O. Besenhard

**Affiliations:** a Department of Chemical Engineering, University College London, London WC1E 7JE, UK; b Department of Biochemical Engineering, University College London, London WC1E 7JE, UK

**Keywords:** Asynchronous operation, High-throughput experimentation, Lab automation, Liquid handling robots

## Abstract

Growing demand for complex and efficient high-throughput experimentation is accelerating laboratory automation, yet liquid-handling robots, which are central to these workflows, remain constrained by sequential operations that limit scalability. To address this bottleneck, an asynchronous scheduling hub system, named Foehn, was developed to enable concurrent and coordinated control of multiple experimental modules within a robotic workstation. The Foehn integrates open-source hardware based on the Arduino microcontroller with a Python-based graphical user interface, forming a flexible and cost-effective control architecture. Besides, it enables asynchronous, multi-threaded operation through standardized serial protocols, managing communication between the liquid-handling robot and peripheral modules such as pumps and magnetic stirrers. Validation tests confirmed stable voltage output from H-bridge drivers, achieving a high pulse-width modulation control efficiency of 87.6%. The integrated Foehn system successfully executed concurrent pumping and stirring tasks while the liquid-handling robot performed pipetting and labware-moving, demonstrating excellent synchronization and operational stability across hardware layers. Combining modular design, open-source accessibility, and precise digital control, the Foehn system provides a scalable foundation for high-throughput automation and holds strong potential to accelerate research in chemistry, biology, and materials science by bridging benchtop setups with next-generation robotic laboratories.

## Graphical abstract

**Figure:**
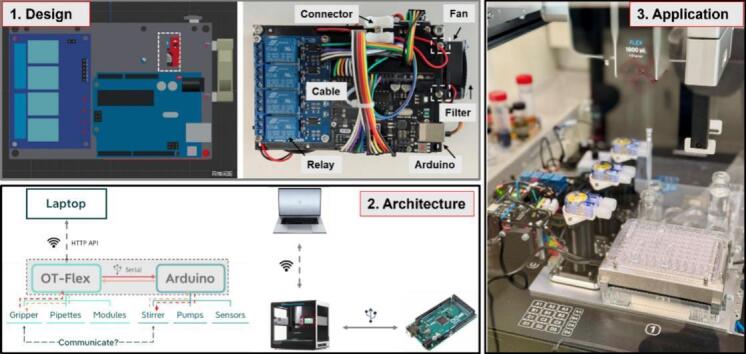

Specifications table.

**Table:** 

Hardware name	Compatible Asynchronous Central Control Node-Foehn
Subject area	• Engineering and materials science
Hardware type	• Mechanical engineering and materials science
Closest commercial analog	• No commercial analog is available.
Open source license	• CC BY-SA 4.0
Cost of hardware	• $250 (Bill of materials).
Source file repository	• https://doi.org/10.17605/OSF.IO/R2PVK

## 1 Hardware in context

Recently, rapid development of liquid handling robots (LHR) has allowed repetitive and tedious tasks gradually replaced by automation, especially in biochemical and pharmaceutical fields where data-driven methodologies are increasingly applied [1], [2], [3]. A variety of open-source and modular robotic platforms have merged, offering compact designs, universal deck layout, and flexible hardware extensions that allow customized workflow [4], [5]. Among these, widely adopted systems such as the Opentrons (OT) series exemplify a versatile and cost-effective solution, hence becoming increasingly popular due to their flexibility in terms of control for academic research [6], [7]. Compared with manual pipetting, the automated procedure offers substantially higher accuracy and precision, significantly improving experimental reproducibility and scalability [8], [9]. Some interesting studies regarding the application of LHR in different research fields also confirmed the tendency [10]. For instance, Chow et al. developed a scalable workflow based on that to explore the bacterial lipases for biodiesel production [3]. Higgins et al. combined LHR with rapid throughput characterization to study the stability of multicomponent lead halide perovskites [6]. Gao et al. used the customized liquid handling platform to investigate the aggregation behavior of nanoparticles [11].

However, as the demand for high-throughput experimentation (HTE) rapidly rises in the screening and discovery of new materials [12], [13], conventional workflows based solely on pipetting functionalities of robots are now reaching their practical limitations. One main restriction arises from the sequential nature of liquid handling operations, where each step must wait for the completion of the previous pipetting action before proceeding. Consequently, the overall workflow efficiency is constrained by the robot’s operation cycle, hindering its applicability to parallel experiments. Previous studies have attempted to mitigate this bottleneck by employing multiple robotic units operating in parallel within the same workspace [10]. However, such an approach inevitably leads to a substantial increase in system cost, as the fundamental issue of sequential task execution remains unresolved. Similarly, multi-channel pipetting with well plates has been explored to accelerate liquid handling operations [7], [14]. Although this approach improves pipetting efficiency, it offers limited overall benefits since subsequent steps such as stirring, mixing, or liquid transfer still need to be executed sequentially. As a result, the workflow remains constrained by the single-threaded operation of the robotic platform, preventing further enhancement in experimental efficiency. This limitation is particularly critical in industrial and semi-industrial high-throughput environments, where continuous operation and parallel execution of multiple unit operations are essential. In such settings, strictly sequential robotic workflows lead to underutilized peripherals and reduced overall efficiency.

Despite significant advances in liquid-handling robotics and modular laboratory automation, most existing platforms focus primarily on improving pipetting efficiency or hardware integration, while the overall workflow remains governed by a centralized, sequential execution logic. Previous studies have demonstrated parallelization through either multi-robot systems or hardware-specific control solutions; however, these approaches often require complex system-level integration, increased cost, or tightly coupled configurations. Therefore, a general and open-source scheduling framework that enables truly asynchronous and decoupled control of experimental modules within the robotic workstation remains largely unexplored. To overcome this traditional bottleneck for HTE workflows with extensive combinatorial trials, the integration of modular extensions within the robotic platform is the key step to enable asynchronous and parallel operations. In typical HTE workflows, multiple separate procedures such as pipetting, stirring, moving and loading of labware often occur repeatedly and independently [15], [16], yet conventional liquid handling robot platforms execute them in a strictly sequential order due to the absence of inter-module coordination. To enable efficient parallel operations, a modular and adaptive control framework is required to manage simultaneous operations, allocate tasks dynamically, and synchronize device communication within the workstation. Based on this concept and requirement, an asynchronous scheduling hub is proposed as the central coordination node to manage communication and task execution between the robotic platform and peripheral functional modules.

In this work, an asynchronous control architecture was developed to enable concurrent task execution within the automated workstation to meet these requirements. The Foehn system was developed using open-source hardware based on the Arduino microcontroller and software framework, which provides high flexibility, accessibility, and cost-effective features. This scheduling hub was designed as the central controller to coordinate communication and operation between the liquid-handling robot and peripheral functional modules. From a hardware perspective, functional modules such as magnetic stirrers, signal light systems or pumping units can act as an independent execution unit equipped with its own microcontroller interface. Using open-source communication protocols and standardized serial interfaces, the central scheduler coordinates task execution and feedback among modules in a synchronized yet decoupled manner. Furthermore, leveraging open-source design principles also simplifies system modification and future expansion, enabling additional devices to be integrated without significant modification. Such distributed and extensible coordination substantially increases resource utilization and enhances overall throughput without the need for additional robotic units.

In addition, the physical structure of the asynchronous scheduling hub was fabricated using low-cost 3D printing technology. The use of polylactic acid (PLA) material not only ensures environmental sustainability but also provides sufficient structural stability for long-term operation. Its compact and standardized footprint facilitates installation and adaptation in various experimental environments, particularly within enclosed or space-limited setups such as robotic workstations. The overall framework of Foehn integrates modular hardware components, open-source communication protocols, and visual software control into a unified automation hub capable of coordinating multiple experimental modules simultaneously. Furthermore, the incorporation of a three-channel signal light system, together with a dedicated graphical user interface (GUI), enhances both functionality and user interaction, forming a cohesive and intuitive control system for real-time monitoring and task management. This integrated open-source design enables rapid, low-cost customization while maintaining flexibility and scalability, facilitating efficient high-throughput experimentation and accelerating scientific research across diverse application fields.

## 2 Hardware description

**Fig. 1:**
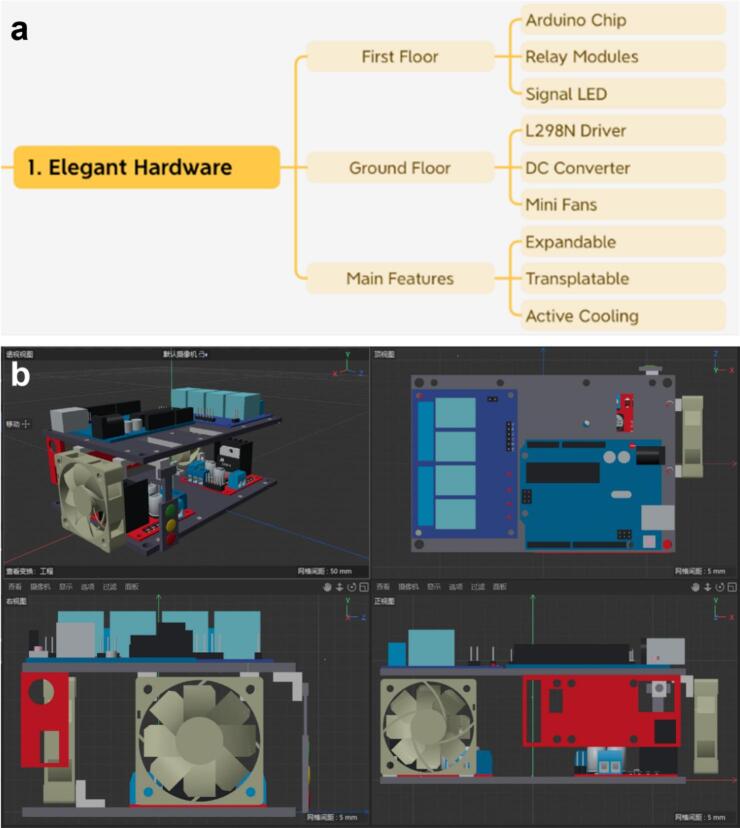
Overall structure of the asynchronous scheduling hub. (a) Mind map including two different layers with electronic modules, ensuring multi-functional features for compatible deployment across different laboratory instruments. (b) 3D-rendered three views provided for a detailed demonstration of its structural layout and functional modules.

The main hardware structure for the asynchronous control node (Foehn) can be divided into 2 floors with a mind map summarized in Fig. 1a. In general, the Foehn scheduling hub system consists of three components: (1) the first floor (top layer), which houses the Arduino microchip and the 4-channel relay module, with a signal light system hanging on the flank side; (2) the ground floor (bottom layer), featuring the same standard ANSI/SLAS footprint (127.76 × 85.48 mm) as the upper level, accommodating two dual-channel L298N drivers equipped with an active cooling system for stable and efficient operation. (3) the supporting structure, composed of aluminum standoffs between the two layers to reinforce the framework, further facilitating the expanded space for maintenance and cable routing.

This compact and unified design, fully compliant with the ANSI/SLAS standard recommended by the Society for Laboratory Automation and Screening, ensures dimensional compatibility with a wide range of laboratory instruments like robotic arms and liquid handling systems, thereby facilitating seamless integration within standardized experimental platforms across different manufacturers. Fig. 1b shows the complete 3D-rendered view from different perspectives, highlighting the structural layout of the top layer (Arduino and relay module), bottom layer (dual L298N drivers with active cooling), and aluminum standoff support structure as mentioned above.

**Fig. 2:**
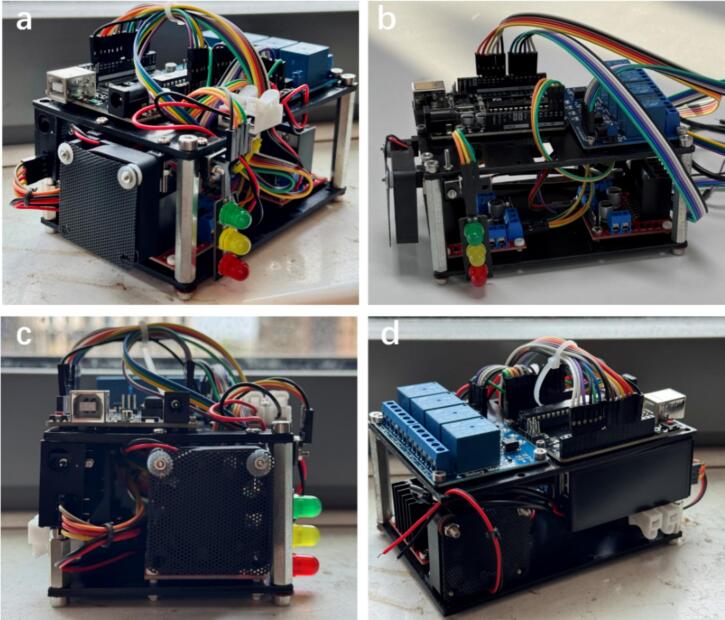
Prototype of the Foehn system, with an AT-Mega microchip and multi-channel control modules integrated into a compact, two-level structure. (a-d) Photographs from different viewing angles illustrating the overall layout and spatial arrangement of sub-sections, aiming to establish the physical architecture of the system.

The final hardware prototype was assembled and displayed in Fig. 2, providing a system-level overview of the physical realization of the Foehn scheduling hub and its correspondence to the 3D-rendered design. Three pictures from different perspectives clearly demonstrate the compact and modular configuration. The internal cabling was organized in the actual assembly for improved accessibility (Fig. 2**a-b**), and convenient JST connectors were added to the output ports of the L298N drivers to facilitate quick connection and output voltage detection, which will be discussed in the following sections. In addition, an air filter was installed for each individual fan to protect the cooling system and ensure its long-term operational reliability, as shown in Fig. 2c. Compared to the rendered model, the actual prototype incorporates quick wiring terminals and a protective power cover lid (Fig. 2d), enabling functional expansion and enhanced system safety for further adaptability and maintenance. Overall, these figures aim to provide a holistic view of the assembled system, while detailed functional modules and performance evaluations will be discussed in the following sections.

### 2.1 Top layer

#### 2.1.1 DC power supply

The first floor serves as the core of the asynchronous scheduling hub system which houses the Arduino microchip with relay modules for multi-channel control functionality. Furthermore, a 12 V DC power adapter was integrated as the general input power supply, with adapted voltages output including 12 V, 5 V, 4.2 V, and 3.3 V for different electronic modules inside.

**Fig. 3:**
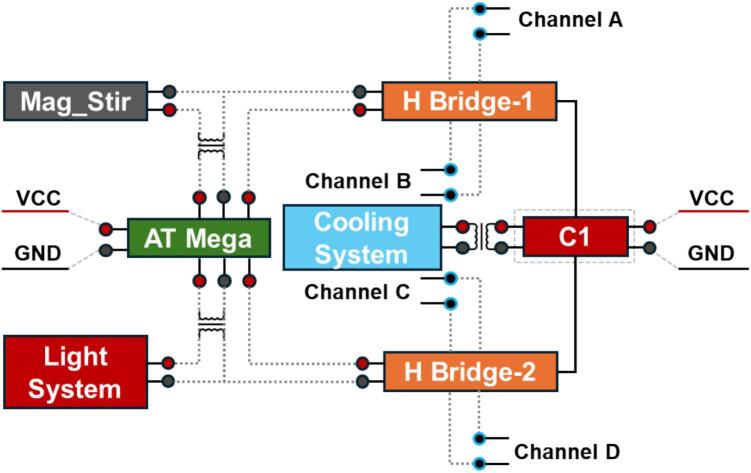
The framework of the power supply system with Integrated I/O for 12 V, 5 V, 4.2 V, 3.3 V devices for all modules.

Considering a series of electronics were integrated into the hub system, the power supply system required a unified input design to simplify its internal framework. As shown in Fig. 3, a brief demonstration of the DC power input system (C1) was designed to fit the above-mentioned features. Located on the flank side of the first layer, it supplies stable regulated power to the Arduino controller, cooling system, relay boards, and dual L298N drivers. It should be noted that the power supply for the magnetic stirrer is independent from the main control module, as the Foehn system only manages its on/off state and operation duration rather than directly providing power or speed regulation. Meanwhile, the two H-bridge driver modules (L298N) support pulse-width modulation (PWM) control of four independent output channels (A to D). Currently, these channels are assigned to drive four external peristaltic pumps, enabling precise and independent flowrate with direction regulation.

#### 2.1.2 Overall labelled structure

**Fig. 4:**
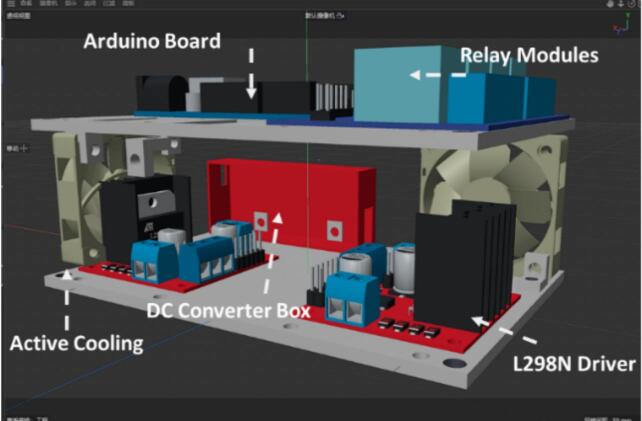
Demonstration of the framework of the asynchronous scheduling hub with labelled modules for functionalities.

An overall isometric rendering of the main structure is presented in Fig. 4, with the functional modules labeled to facilitate clearer understanding of the layout. It should be noted that the L298N driver board can generate joule heat while driving the pumps with PWM control, although the driver board itself has the heat sink fins, poor thermal management still restricts its long-term performance. A vast amount of the heat generated from the electronic components not only interferes with the operation of adjacent electronic modules but also can be a hazard (e.g., burning the skin) [17], therefore active cooling system is designed to solve this problem.

Two independent DC fans were integrated into different levels to maintain a hybrid airflow across the whole device, ensuring uniform heat dissipation and stable operation. A detailed discussion for the airflow will be included in the next section based on the bottom layer structure.

### 2.2 Bottom layer

#### 2.2.1 Active cooling system

**Fig. 5:**
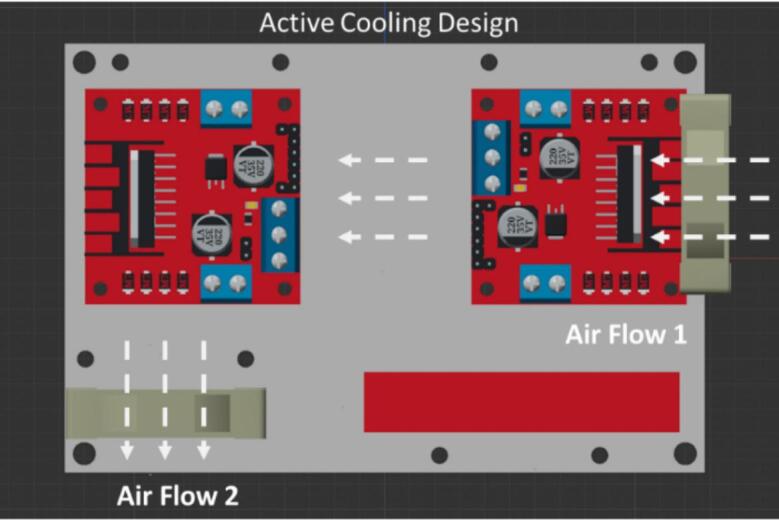
The hybrid cooling architecture of the Foehn with two vertical airflows inside for heat dissipation.

The ground floor accommodates the two driver modules as well as the space for cable connections. Therefore, the issue of heat accumulation in this confined area must be carefully addressed. Fig. 5 demonstrates the airflow direction of the Foehn, ensuring a circulating air environment while the operation of driver systems. The integration of the vertical cooling architecture solves the thermal management problems and greatly increased the efficiency and safety of the driver board in the long-time running application. Apart from the cooling of the L298N boards, airflow 1 of the cooling architecture also takes the thermal management of the DC converter chip into account, as the side flow can help relieve the small amount of accumulated heat from the flank of top layer.

#### 2.2.2 Main plates and extension design

The overall design of the distribution concept is to put the high voltage modules on the ground floor while leaving the control modules with low voltages like microchips and relays on the first floor. Flexible holes for M3 and M4 are designed on both mainboards to ensure the future extension for any upgrade maintenance. The fixing method of the cooling fan was based on the M2 with unique hinge accessories on different levels, realizing the minimum footprint with a fixing vertical direction. Therefore, leaving extra space for any cables and extended ports on the first floor.

### 2.3 Supporting structure with expandable features

#### 2.3.1 Aluminum standoffs

To optimize the overall structural strength relative to weight, a balanced design combining 3D-printed plates and aluminum standoffs was adopted. The main supporting structure is both simple and reliable, as the standoffs are positioned at the four corners of the PLA-printed plates on both layers to ensure mechanical stability and uniform load distribution. Furthermore, the vertical accessories (3D-printed) for fixing DC fans were also optimized to save extra space without occupying adjacent area after integration with robotic platform.

#### 2.3.2 Signal light system

**Fig. 6:**
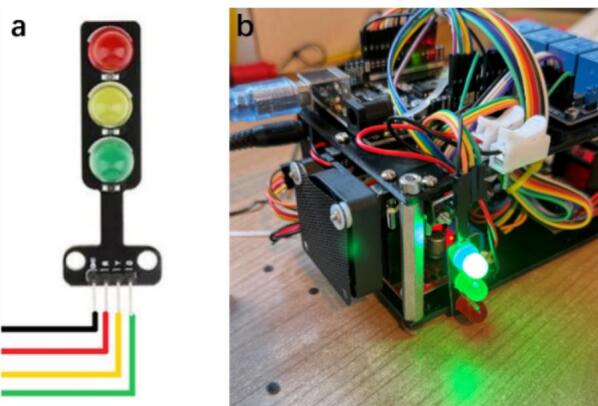
(a) Illustration of the LED signal module with red, yellow and green indicators. (b) Experimental demonstration of the LED signal light system providing real-time visual feedback for module status. (For interpretation of the references to colour in this figure legend, the reader is referred to the web version of this article.)

The signal light system inside is used to display the current running status of the Foehn, making the monitoring process easier during the lab-automation. As shown in Fig. 6a, the LED signal light with red, yellow, and green was used here for different purposes. For instance, the green light represents the working condition of the pumping system, indicating that at least one channel of the L298N driver is operating. Therefore, users can immediately know the scheduling hub system is controlling the pumping channel (Fig. 6b). The yellow light serves as an indicator for the magnetic stirrer, which remains illuminated whenever the stirrer is in operation. The red light is reserved for system alerts or error notifications with wrong input command, providing a clear and intuitive visual cue during operation.

Notably, the on/off state of each LED is synchronized in real time with the corresponding hardware module activity, exemplifying the multi-threaded control capability of the asynchronous scheduling hub system. This design offers an effective physical visualization of the system’s asynchronous operation and status indication.

#### 2.3.3 Expandable ports

**Fig. 7:**
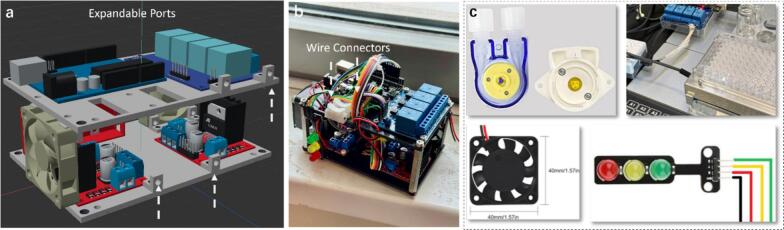
(a) Demonstration of the expandable ports and spare space for potential upgrade applications. (b) Current use of extended ports with wire connectors for cable organization. (c) Integrated control of multi-channel modules within a single hardware (Foehn) via a systematic solution with GUI.

Fig. 7a shows the extended interface design, which was developed to allow future upgrades and modular expansion in the future. These 3D-printed accessories, equipped with compatible screws, can be used to mount small auxiliary modules or connect additional standard ANSI plates, thereby enabling customized applications. Currently, those ports are used for cable management with connectors and reserved slots planning within the structure, as presented in Fig. 7b.

In summary, with the optimal design based on the structural framework via aluminum columns and Polylactic Acid (PLA) materials, several promising properties including flexibility, affordability, and compatibility were achieved. In a research laboratory application scenario (Fig. 7c), the Foehn scheduling hub system can be used to:

• Realize asynchronous operation with functional control of multi-channel modules via a compatible GUI.
• Integrate with a robotic platform for high-throughput experimentation (HTE) with asynchronous control for lab-automation in the multidisciplinary research fields.
• Study the operating characteristics of DC Pumps with high precision on digital PWM Flowrate and direction control.
• Investigate the impact of stirring parameters on the reaction process by controlling the magnetic stirrer system for novel materials discovery.
• The versatile and open-source design with GUI not only enables adaptable compatibility to various lab-equipment manufacturers but also acts as a good teaching tool for lab-automation courses.

## 3 Design files summary

The design files listed below include all documents required to construct and assemble the Foehn scheduling hub system, with necessary images for guidance. These files include 3D-printable components, reference images, robot node firmware, a graphic user interface, and a bill of materials. All those files mentioned above are available on the repository link (https://doi.org/10.17605/OSF.IO/R2PVK).

**Table:** 

**Design file name**	**File type**	**Open source license**	**Location of the file**
**Split Single Parts.zip**	CAD Files	CC BY-SA 4.0	
Floor-1.stl	STL	CC BY-SA 4.0	
Floor-2.stl	STL	CC BY-SA 4.0	
Insert lid for box case.stl	STL	CC BY-SA 4.0	
Small Box case.stl	STL	CC BY-SA 4.0	
Small Brackets.stl	STL	CC BY-SA 4.0	
**Overall Structure**	Fig. 1, Image	CC BY-SA 4.0	
Assembled Floor-1.jpg	Image	CC BY-SA 4.0	
Signal Light System	Fig. 6, Image	CC BY-SA 4.0	
Cooling System.jpg	Image	CC BY-SA 4.0	
Modelling Design.png	Image	CC BY-SA 4.0	
4-Channel Relays.jpg	Image	CC BY-SA 4.0	
Valdiation Test 2.mov	Video	CC BY-SA 4.0	
**RobotNode_Firmware.ino**	Arduino IDE	Open-source license	
RobotNode_GUI_Win.py	Python	Open-source license	
RobotNode_Test.py	Python	Open-source license	
BOM.xlsx	Excel	Open-source license	

**Split single parts** refer to the components fabricated on the 3D printer and the CAD files localization, with editable design files included. The individual STL files for constructing the prototype were listed below the zip package. It should be noted that the accessories (small box case and the insert lid) were in combination parts. Floor-1 and 2 represent the main plate of the bottom layer and the top layer, respectively.

**Overall structure** of the three-views of the final product was listed and included here as an assemble guidance, with detailed explanation under the previous chapter. The assembled floor image was given to show the aluminum columns with supporting structure. Image of the cooling system presents the assemble direction guidance for the DC fans, with the modelling design figure as a complement.

**RobotNode Firmware** is the Arduino code used for controlling the Foehn scheduling hub system with multi-thread functionalities. RobotNode GUI is a graphic user interface designed to operate the Foehn based on the python script for windows system, users are required to install some basic python dependencies to run the application. The detailed bill of materials was summarized in an Excel sheet as shown in the table.

## 4 Bill of materials summary

The majority of the components used in constructing the hardware device were easily obtained either from amazon website or in the lab at a low cost. The whole body can be divided into 4 categories including main electronic modules, accessories, 3D-printed items, and assembling items. The primary cost was the purchase of those electronic modules with accessories for realizing control and driving functionalities. Apart from that, rest of the components were optimally selected and used for assembling connections at a relatively low budget. Therefore, the total cost of this device is minimized for most lab-based research teams. Some of the supporting accessories were 3D printed with the FDM printer, using PLA filament as the source material for a sustainable and cost-effective method. Since the main structure of this device was based on the printed plates with long metal screws, the fabrication cost for the main body was greatly reduced by avoiding traditional CNC or laser cut manufacturing approaches. For the final prototype of the Foehn device, the total weight was controlled under 0.8 kg, resulting in flexible and portable application features for different scenarios. The complete bill of materials (BOM) can be found on the repository (https://doi.org/10.17605/OSF.IO/R2PVK). Furthermore, an Excel document included a detailed description of the components, material sources, and their approximate costs were summarized for reference.

## 5 Build instructions

The assembly process of the Foehn system is easy and convenient due to the structural design of each level; all components are secured using M2 or M3 except for the aluminum columns with M4 screws. Therefore, no adhesive is required during the construction. In brief, the assembly process can be divided into three stages: (1) attach the Arduino microchip and 4-channel relays on the top layer; (2) fix dual L298N drivers with the cooling system on the bottom layer then connected to the DC converter; (3) assemble the aluminum standoffs on the four corners with supporting accessories then adjust the signal light module. Finally, all components of Foehn are attached together within the main structure.

### 5.1 Top layer construction

#### 5.1.1 Main electronics

**Fig. 8:**
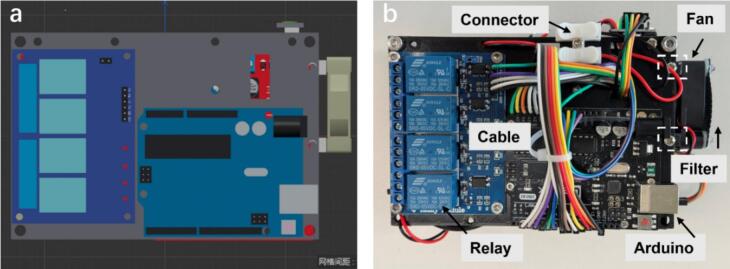
(a) 3D rendering of the top layer of the Foehn scheduling hub system. (b) Assembly instruction of the top layer.

During the first stage, the key components are the Arduino UNO microchip and the 4-channel relays module. From the left part shown below (Fig. 8a), the construction of top layer is based on the customized ANSI board which refers to the Floor-1.stl file mentioned in the design file section. The screw holes are designed for the standard size of the Arduino UNO, but they are compatible with different manufacturers since the attachment can be adjusted using the M2 screws with corresponding nuts. Similarly, the relay module can be secured onto the top layer using M3 screws and nuts. It is recommended to add spacers of matching size during installation to ensure proper alignment and stability. A photograph of the assembled components in the top layer is provided in the design files named Assembled Floor-1.jpg.

#### 5.1.2 Cooling system and accessories

Caution should be taken when assembling the wire connector across the M3 screw hole. The matching nut should not be overtightened, as the connector needs to remain flexible and able to rotate. This is reserved to check the signal from Arduino I/O board for testing and organizing the nearby cables. It should be noted that the assembly of cooling system on the top layer requires the use of 3D-printed accessories (Small Brackets.stl) which can be found in the design files summary. This printed component is a vertical adapter bracket which is compatible with long M2 screws (16 mm) for attaching the DC fan from the flank side without occupying the space on the main board. A pair of small brackets which obtained from the 3D-printing for attachment are marked with dashed line box in Fig. 8b. The DC 4010 cooling fan can then be fixed using M2 screws with nuts across the holes inside the brackets accordingly. A special note is that an air filter accessory is recommended to attach in front of the DC fan, ensuring the safety and stable operation of the cooling system in the long term. A typical assembly procedure is to place two M2 spacers above the fan’s screw holes, which creates a small gap between the fan and the filter to allow sufficient airflow intake, followed by fastening with matching nuts.

#### 5.1.3 Electronics wiring

**Fig. 9:**
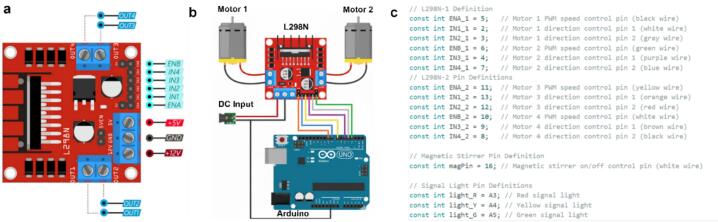
(a) Illustration of the interface definition for the L298N driver board. (b) Typical wiring and interface connections for control modules and peripheral devices with motors and Arduino microchip. (c) Demonstration of the detailed mapping connections for hardware interfaces listed in the firmware code.

The connection of Arduino board with sub-modules follows specific rules, where each I/O port is assigned to a unique and well-defined functionality. Fig. 9a shows the interface definition of the L298N driver board, where PWM-enabled pins are mapped to the enable (ENA/ENB) inputs, while dedicated digital pins control motor direction. This configuration reflects the functional separation between speed modulation and directional control. As shown in Fig. 9b**, a** detailed wiring schematic is provided for the dual-channel driver board as reference for reproduction. In this configuration, the Arduino microcontroller acts as the central control unit, generating both PWM signals and digital direction commands. These signals are transmitted to the L298N driver, which subsequently regulates the operation of independent DC motors. Specifically, the pins (IN1–IN4) are connected to standard digital outputs to define the motor direction. The L298N thus functions as an intermediate power amplification and control stage, translating logic-level signals from the Arduino for driving external motors.

The detailed definition of each interface (including pin number, signal type) and its corresponding module connection are listed in the comments of the RobotNode Firmware file (Arduino IDE), which can be found in the design files section. These definitions are documented as in-line comments (Fig. 9c), serving as the wiring reference for the system and ensuring its reproducibility. By embedding the wiring logic directly into the firmware, both hardware reconstruction and future modification are significantly simplified. The general wiring rules and cable management follow a hierarchical principle, meaning that each module is connected to its nearest interface to minimize cross-wiring while maintaining a balanced load across the power interfaces from the Arduino port. A special note is that the signal wires of the relay module should not occupy the PWM pins of the Arduino, as these pins need to be reserved for the L298N motor driver on the bottom layer for PWM control. Due to the double-layer design, the Dupont wires connecting to the modules on the bottom layer are routed through the reserved rectangular openings. This arrangement not only keeps the wiring layout neat but also enables efficient inter-layer communication with the hardware on the bottom layer.

### 5.2 Bottom layer construction

#### 5.2.1 H-bridge drivers

**Fig. 10:**
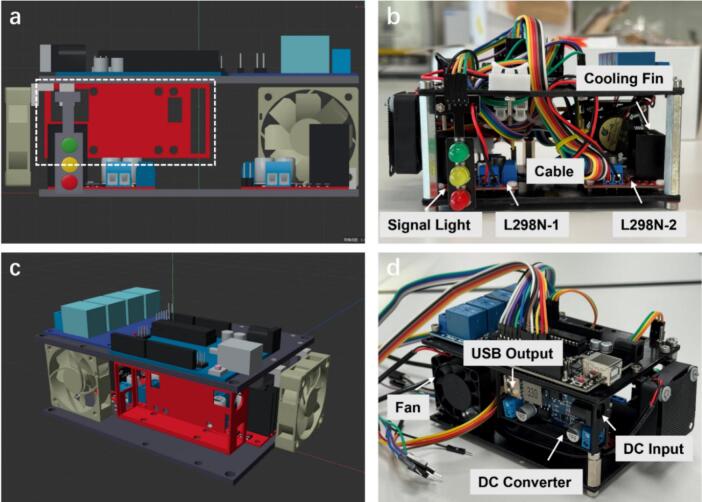
(a) Side-view 3D rendering of the Foehn scheduling hub system. (b) Detailed assembly instruction of the H-bridge drivers on the bottom layer. (c) Perspective 3D rendering of the Foehn for demonstration of the power converter. (d) Detailed assembly instruction of the integrated power converter with 3D-printed protective accessories.

As shown in Fig. 10a, the dual L298N drivers are attached with M3 screws on the bottom layer, with screw holes reserved for easily fixing their positions. It is recommended to use nylon screws and nuts to prevent potential short circuits, since the power input is designed to drive dual motor channels for each module. Apart from that, the negative power input of the L298N drivers must share a common ground with the Arduino’s GND to ensure stable signal transmission. Therefore, a shared Dupont wire is required to connect from the bottom layer to the GND port on the top layer through the reserved rectangular opening. It should be noted that the two drivers are labelled in advance to avoid wrong connection with the Arduino board, as the Dupont wires extended from the top layer are reserved for the two modules with specific interface (Fig. 10b). The power supply of the L298N drivers comes from the DC input, which shares the same voltage as the converter input. Caution was taken when designing the cable management, as the temperature of the cooling fin may be high during the operation. Therefore, the upper space of the L298N driver is suggested to be reserved for the heat dissipation, preventing any contact with wirings or connectors.

#### 5.2.2 DC power converter

The power supply is a key component of the Foehn system, as different modules require distinct operating voltages. Therefore, the assembly of the DC power converter should be carried out carefully to ensure reliable voltage regulation and stable operation. As shown in Fig. 10c, the perspective view of the rendering image clearly demonstrates the location of the power converter, hanging on the flank side between the two layers using the shared M2 screw hole with the Arduino board. A special note is that to strengthen the framework and protect the converter from any dust, the 3D-printed accessories (Small Box case.stl) are manufactured to fit the converter module with a cover lid. Several M3 screw holes are reserved on the backward of the box case for the attachment. Therefore, the power converter can be fixed in a reliable way as illustrated in Fig. 10d. A general AC-DC power adapter (12 V) is used as the main power supply, which can be easily obtained from any mechanical workshop or online electronic stores. A round socket is integrated into the converter box to accommodate the DC input plug. It is recommended to operate with caution to avoid applying excessive force that may damage the protective casing or the power components.

The DC converter provides a 5 V output through two interfaces: a USB port and a blue terminal block. The control modules receive power from the terminal block at present, as they are compatible with multiple Dupont pins across different layers. The remaining USB output is reserved for potential modular plug-in upgrades to supply power to future components. The cooling system in the bottom layer shares a similar assemble method compared to the top layer, as the fan is first attached to the 3D-printed brackets to form a small gap between the bottom plate. Furthermore, this space allows the output connection of the second L298N driver, optimizing the space and footprint of each electronics inside. A complete reference image of the cooling system can be found under the design files summary (Cooling System.jpg).

### 5.3 Main framework construction

After the assembly of electronic modules on two layers of the Foehn, the construction of its main framework is considered as the final stage. To ensure the overall structural strength of the hardware device, aluminum columns (70 mm) with internal M4 threads are selected as the supporting components. As shown in the previous image (Fig. 10b), the aluminum standoffs are attached at the corner of the plate between the two layers. To ensure structural stability, additional washers and M4 nuts are used for secure fastening on both sides of the standoff.

It should be noted that a shorter 20 mm aluminum standoff is used on the side of the power converter, as part of the vertical clearance is occupied by the protective casing (Fig. 10d). This design ensures both mechanical stability and spatial compatibility between the converter housing and the overall framework. It’s suggested to check the designator columns in the Bill of Materials sheet, as it also provides the necessary description for assembling components with 3D-printed accessories. To complete the assembly, the signal light module is required to be attached vertically to the side of the main framework. A cover lid can be inserted into the protective case of the power converter as an option. After organizing the cables and Dupont wires with nylon ties through the reserved openings inside the top layer, the final hardware assembly resembles a compact rectangular container with a neat and orderly appearance, as shown in the design file (Modelling Design.png).

## 6 Operation instructions

The Foehn scheduling hub system is easy to operate once the assembly is completed following the procedures. Start by downloading the RobotNode Firmware from the repository, then open the software code in the integrated development environment of Arduino. There is no need for the modification of the firmware as long as the construction process is accurate. Connect the Foehn system with laptop via a USB cable and then upload the code to the board to complete the basic setup. Ensure that no extra input is connected to the hardware device at this step during the upload process. After that, place the Foehn system at a desired horizontal location for initialization. Before connecting to any external modules (e.g., DC pumps), the AC-DC adapter should be plugged into the reserved interface of the power converter, serving as the power supply for the active cooling system and extended electronics. Besides, using the GUI application is recommended for controlling and monitoring the feedback of the Foehn system, which can be downloaded from the repository (RobotNode GUI). It should be noted that the GUI is designed to run on the Windows 10 and 11 platform, as the application is developed using Python’s tkinter framework with related dependencies. An integrated development environment (IDE) such as Visual Studio Code (VS Code) can be used to modify, debug, or execute the program when further customization is required.

### 6.1 Initialize the Foehn node

The first step is to ensure all the cable connections have been completed and plugged in, including the power supply system. Make sure that the output voltage of the AC-DC adapter is aligned with the properties of DC pumps or other extended electronics. Customized ports can be attached for convenience when necessary. In the current version, SM ports are selected as the output connections for easy plug in and out options, a 12 V output voltage is used for driving the pumping systems. Remember to double check the connection of Arduino UNO USB cable, as further system communication and data sync all rely on this part.

### 6.2 Setup the graphic user interface

**Fig. 11:**
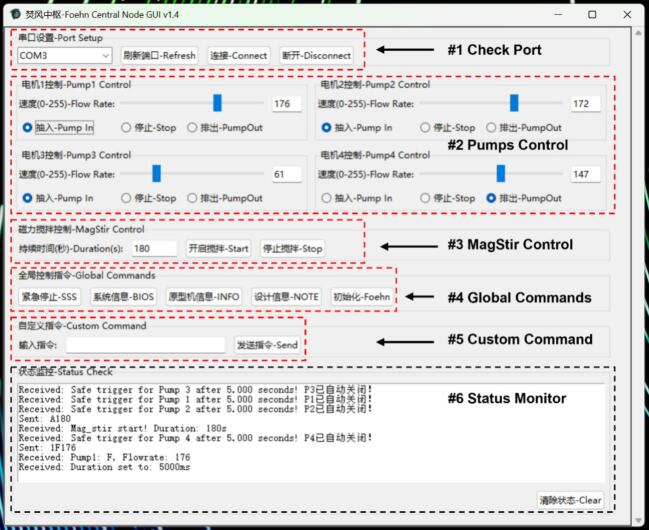
The stable version of Foehn GUI for Windows system, while the core framework is based on the tkinter (Python).

Open the GUI application under the VS Code IDE and then run the script at the Windows platform to ensure its functionality. The related Python packages which can be accessed free of charge, are required to install to realize the full functionality of the GUI. The application was developed using Python 3.11.9 (Win 10 and 11) with the built-in tkinter library for GUI construction and the threading module for multi-threaded execution. Serial communication was handled using the pyserial library (v3.5). All other functionalities rely on Python standard libraries (os and sys), ensuring minimal external dependencies and high reproducibility across different laboratory environments. Once the basic dependency has been completed and is ready to use, users can run the application and then enter the GUI as shown in Fig. 11.

Before operating, it is important to check the port configuration. Normally, the GUI will automatically detect all available COM ports connected to the computer and display them in the dropdown menu. If not, users can click the refresh button to get updated information and then connect to the Foehn system by clicking the related button on the GUI. Once the first step is done, it’s quite easy to control and operate the Foehn scheduling hub system to realize asynchronous operation using the sub-sections below.

### 6.3 Control the 4-channel pumping system

The second part of the GUI (marked with red dashed line box) is used to control the 4-channel DC pumps, under each sub-block there are two parameters that can be defined by the user: the pumping flowrate (0–255, corresponding to the PWM value) and the operation direction (in, out, or stop). The flow rate can be specified either by directly entering a value via the keyboard or by adjusting a slider bar for intuitive control. Furthermore, the four DC pump channels can operate simultaneously without interfering with each other. Each channel maintains independent state and flow rate control, enabling fully asynchronous operation of the pumping system.

### 6.4 Control the magnetic stirrer system

The magnetic stirrer used in this setup was the MIXdrive 96 MTP (2MAG, Germany) for 96 well microtiter plates (technical details can be found in BOM). The control panel for the magnetic stirrer is in the third section of the GUI, where two parameters can be defined by the user: the operation duration (in seconds) and the operating status. It should be noted that the user can stop the stirrer even if a previous command (e.g., stirring for 60 s) is still being executed. This is made possible by the multithreading mechanism integrated into the Foehn system, which allows new commands to overwrite previously issued ones, providing greater flexibility and responsiveness during operation.

### 6.5 Send global and custom commands

The fourth section of the GUI contains several predefined buttons that correspond to global commands. These commands are particularly useful in emergency situations or for checking the basic status of the Foehn system. For instance, the “SSS” button serves as an emergency stop. Once the user clicks this option, all modules and electronic components are immediately powered off to ensure operational safety. This functionality is especially critical when a reaction process is underway but must be interrupted due to potential system errors or unexpected conditions. The ‘BIOS’ and ‘INFO’ button provides self-checking functionalities which can be used to check the firmware version and the hardware identification. Furthermore, the ‘Foehn’ button can be used to refresh the status of the hardware device, once the user clicks the button, the Foehn system will shine with all signal lights for 3 s while shielding all other commands as an indication, this is normally used during the initialization with robotic workstation for visual confirmation.

Because the Foehn system is designed to integrate with the robotic workstation, hence the bottom controlling commands follow a specific rule therefore can be sent via Python script. If users are familiar with those commonly used command strings, it’s also feasible to enter the command line directly into the custom command panel.

The bottom section of the GUI functions as the status monitor for the Foehn system, where users can conveniently review the history of issued commands and the corresponding feedback information from the hardware. This section also serves as a visual communication bridge between the user and the Foehn scheduling hub system, particularly useful for verifying the execution status of specific operations such as the pumping action and flow rate values. Furthermore, any incorrect commands or error statuses can be displayed in this section instantly, allowing users to effectively monitor the running status of the Foehn system. This feature enhances the user-friendliness and reliability of GUI during operation.

### 6.6 Exit GUI and disconnect the Foehn system

Once the operation or testing procedures have been done, a correct sequence of disconnections should be followed to minimize any potential damage to the hardware part. Users can firstly click the disconnect button on the top section of GUI and then exit the application. Besides, it’s always recommended to check the running status of any extend hardware (e.g., DC pumps) and remember to click ‘SSS’ button to shut down and double check the disconnection. After that, unplug the DC power input and other connection ports carefully and then place the Foehn at a horizontal surface to avoid any collisions.

### 6.7 Troubleshooting and maintenance

If the Foehn system fails to receive commands correctly or does not display the proper running status, the issue may be caused by a disconnection of the USB cable between the Arduino and the operating terminal (computer or robotic workstation). To solve this problem, it’s recommended to replace the data sync cable and then firmly connect to the Foehn system again to run the test script (RobotNode_Test.py) in the design files summary. Alternatively, the port configuration can be refreshed through the GUI application discussed above. If any garbled texts (or error messages) appear during the operation, check the decoding configuration in the Arduino IDE. The compatible character encoding for most cases is UTF-8.

If the problems related to the control of running modules, e.g. DC pumps or extended electronics, turn off the device and disconnect the DC power supply first and then restart and connect the hardware to the computer again to check the status. Normally, this problem can be solved following the troubleshooting procedures. However, if the same issue persists, refresh the software of the Foehn system by uploading the original version of the firmware to the Arduino board. In this case, most potential issues can be resolved as the stable firmware has been updated inside.

It should be noted that this device is not designed to withstand mechanical loads or stress, as the overall framework primarily consists of PLA components supported by aluminum standoffs. In case maintenance or replacement of components is required, always disconnect the power supply before handling any electronic parts. In addition, the air filter in front of the DC fan should be cleaned or replaced periodically to maintain efficient airflow and prevent dust accumulation, which helps ensure the long-term reliability and stable performance of the dual H-bridge drivers. The interior space of the Foehn should be cleaned to avoid dust accumulation, as excessive dust may lead to electrical short circuits over time.

To prolong the lifespan of the hardware, it is recommended to operate the device exclusively in indoor environments, avoiding exposure to direct sunlight or any chemically corrosive environments. PLA, the main structural material, tends to degrade rapidly under extreme conditions, such as intense light or high humidity. Alternatively, to enhance long-term durability, the components can be reprinted using more robust materials such as ASA, nylon, or polycarbonate. In this case, only the printing parameters need to be adjusted, with no modification to the design geometry required.

## 7 Validation and characterization

The Foehn scheduling hub system was originally designed for the robotic workstation to realize asynchronous operation during the high-throughput experimentation, but it’s also compatible with multiple operation terminals including personal computers or other liquid handling robots (based on Linux). In this section, two validation tests were performed to characterize the application feasibility and compatibility across different terminal devices as examples. The first test of pumping system was run with customized setup for investigating pumping characteristics with flowrate calibration; this was based on a windows laptop for initial validation of the GUI as well as the multi-thread properties of the Foehn. After integration with the customized robotic workstation (based on the OT-Flex), the asynchronous operation test for HTE was evaluated, hence demonstrating its versatile application characteristics with different scenarios.

### 7.1 Pumping test based on the laptop

**Fig. 12:**
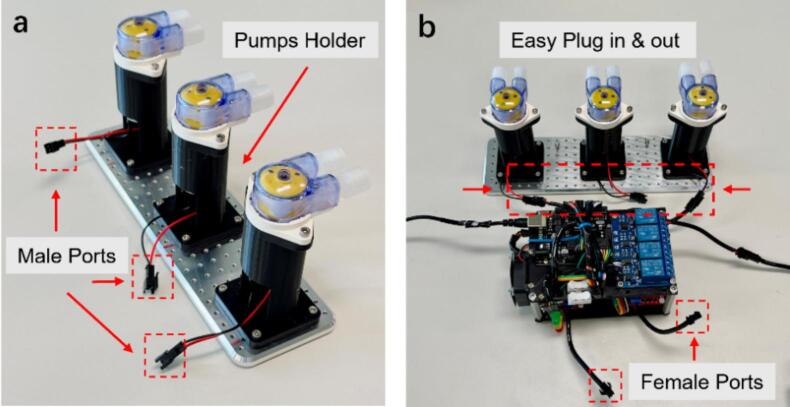
(a) Illustration of the testing setup for the 3-Channel pumping system with male ports. (b) Demonstration of the easy connection with the customized pumping system for the testing setup.

Three peristaltic DC pumps were selected during the first test. To simplify the connection procedure of the Foehn, male ports were fixed via iron soldering as shown in Fig. 12a. A customized 3-Channel pumping system with 3D-printed holder was manufactured as the testing setup. The attachment of those ports greatly increases convenience as the easy plug in and out property is useful during the actual application. For instance, not only the four output interfaces from the L298N drivers can be connected easily with extended devices but also the control channel from the relay module is beneficial, as demonstrated in Fig. 12b.

**Fig. 13:**
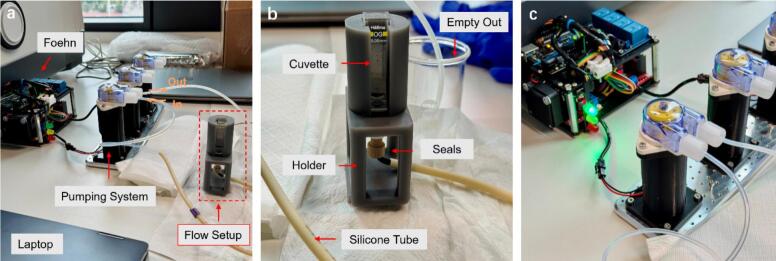
(a) Schematic illustration of the pumping system test with labelled instruction. (b) Magnified illustration of the customized flow setup, details in future work section. (c) Successful validation of the whole setup for the pumping test with correct running status indication from the signal light system of the Foehn.

This test aims to evaluate the driving performance of the internal L298N modules, therefore three different DC pumps (KPRP20, KEF, and ELP02) were studied during this stage. The former two candidates are peristaltic pumps while the last one belongs to the diaphragm pump, these pumps are commercially available from online stores or mechanical workshops and were selected merely to represent different operating conditions. Detailed information can be found on the sheet of BOM. Fig. 13a demonstrates the typical setup during the pumping system test where KEF pumps were used as an example. The test setup mainly includes four parts: the Foehn system, the pumps, the laptop and the customized flow setup. During this period, all the testing tasks were carried out via the GUI discussed before, therefore the laptop was connected to the Foehn with the USB data sync cable. A 12 V AC-DC adapter was used as the power supply for the setup, where the input was plugged into the power case on the flank side of the Foehn system. During this test, users can operate the GUI on the laptop to send corresponding pumping commands to the Foehn. Once the commands are correctly received and executed, the Foehn drives the pumping system to discharge water from the flow setup into a nearby beaker. A magnified illustration with labelled instructions is presented in Fig. 13b as visual explanation.

To explain it further, 2.0 ml water was pre-filled into the cuvette before the testing protocol. The cuvette was placed within a 3D-printed holder inside the setup with a notch design to check the water level. Seals with silicone tube were assembled to ensure the whole circulating system could be run with safety. The test not only includes the pumping operations of different DC pumps but also involves detailed PWM control for further characterization. Fig. 13c shows the running status of the testing setup: the Foehn system correctly received the pump-out commands from the user and subsequently drove the DC pump to discharge water into the beaker as expected. The illumination of the green indicator light confirms the successful execution of these commands, demonstrating that all operation and control procedures were carried out correctly.

Furthermore, due to the easy plug in and out property, different candidates were tested under the same setup to study the characteristics between the peristaltic and diaphragm pumps. The maximum flowrate test was carried out subsequently to validate the rated specs from the manufacturers. Herein, the PWM was adjusted to the maximum value of 255 via the GUI application to compare the flowrate differences. A multimeter was also used to check the output voltage from the L298N interface to ensure that the testing protocol followed the correct principle. Parallel experiments were carried out using the same setup mentioned above, with summarized results displayed in Table 1 for comparison. Those testing results also demonstrated great compatibility and successful control of the pumping system via GUI of the Foehn.

**Table 1:** Results comparison for the pumping system test among three candidates.

**Pump** **Model**	**PWM** **Input / (Hz)**	**Theory Output / (V)**	**Actual Output / (V)**	**Flowrate / (cm^3^ min^−1^)**	**Filling** **Time / (s)**	**Rated Value/** **(cm^3^ min^−1^)**
KPRP20	255	12.00	10.60	42.00	2.86	50
KEF	255	12.00	10.50	93.60	1.28	85
ELP02	255	12.00	10.42	834.00	0.14	600

It should be noted that the actual output voltage from the L298N drivers for all three pump candidates remained highly stable (10.40–10.60 V), confirming the reliability of the Foehn scheduling hub system in precisely controlling the H-bridge drivers. Further investigation on the step control of PWM was also carried out to study the operating properties of different pumps, with flowrate values calculated following a similar approach. The same setup was applied again for the following characterization of the calibration curves among different DC pumps. During this stage, a series of pulse width values was tested via the GUI application to drive the corresponding pump in order to obtain its full operating properties. A special note is that different pumps have distinct threshold value of PWM, this was due to the difference of their motors inside during the manufacturing. Table 2 summarized calibration results of the KEF pump, where the volumetric output was characterized as reference. For each PWM setting, three independent flowrate measurements were conducted to evaluate the volumetric accuracy. The average flowrate was then calculated accordingly to determine the corresponding filling time for a fixed target volume.

**Table 2:** Summarized results for the calibration test of volume accuracy of the KEF pump.

**PWM** **Input / (Hz)**	**Theory Output / (V)**	**Actual Output / (V)**	**Flowrate-1/ (cm^3^ min^−1^)**	**Flowrate-2/ (cm^3^ min^−1^)**	**Flowrate-3/ (cm^3^ min^−1^)**	**Average** **Filling Time / (s)**
120	5.65	5.40	31.20	30.60	31.50	3.86
130	6.12	6.16	40.70	41.30	41.00	2.93
140	6.59	6.71	47.40	47.00	46.80	2.55
150	7.06	7.24	53.70	54.20	54.60	2.22
160	7.53	7.62	57.60	58.30	58.50	2.06
170	8.00	8.03	63.00	62.60	62.00	1.92
180	8.47	8.39	66.00	65.70	66.30	1.82
190	8.94	8.71	70.40	70.80	71.20	1.69
200	9.41	8.95	74.40	76.80	76.20	1.58
210	9.88	9.19	77.80	78.40	77.80	1.54
220	10.35	9.42	82.00	82.80	82.20	1.46
230	10.82	9.61	84.00	84.60	85.00	1.42
240	11.29	9.81	87.60	88.10	87.50	1.37
255	12.00	10.50	93.60	94.20	93.80	1.28

The relatively small variation among the repeated measurements at each PWM level confirms good repeatability of the pumping performance, indicating that the pump can be reliably operated under PWM control for quantitative liquid handling tasks. Based on the summarized results, the filling time required for delivering a fixed volume decreases consistently with increasing PWM input, further validating the predictability of the calibration curve.

**Fig. 14:**
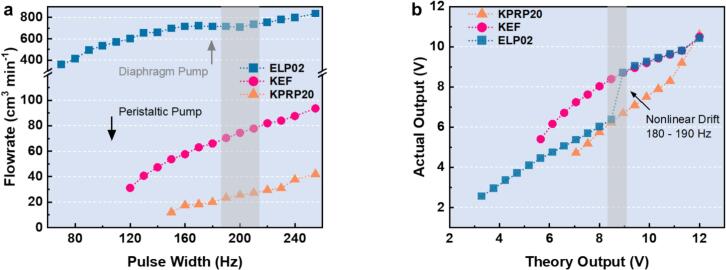
(a) Calibration curve of different pumps via PWM control by the GUI application of the Foehn. (b) Voltage comparison for theoretical and actual values during the test.

Fig. 14a shows the calibration curves of different pumps under a full operating range of pulse width values. It can be found that the diaphragm pump holds a high flowrate around 360 cm^3^ min^−1^ even at the lowest pulse width value of 70 Hz. In comparison, the KEF peristaltic pump has a minimum flowrate around 31.2 cm^3^ min^−1^ at the pulse width value of 120 Hz while maintaining a linear increase under the range of 200 Hz. Interestingly, the KPRP20 pump has the lowest flowrate at each step compared with other pumps, with a highest starting threshold of PWM at 150 Hz. Thanks to the GUI application of the Foehn system, the hardware part along with the software section can be integrated into an organic framework in actual testing application. A detailed output voltage comparison was also done to reflect the characteristics for the pumping control system of the Foehn device. As shown in Fig. 14b, the actual output values of voltage were recorded during the test for different pumps. A nonlinear voltage drift of the diaphragm pump was observed in the range of 8.3 to 9.0 V (corresponding to the 180–190 Hz), indicating an abrupt change of operating property of the ELP02 pump. In contrast, the two peristaltic pumps show relatively mild voltage curves during the test, especially the KEF pump displays good and stable performance under the voltage range from 5.4 to 10.5 V.

**Fig. 15:**
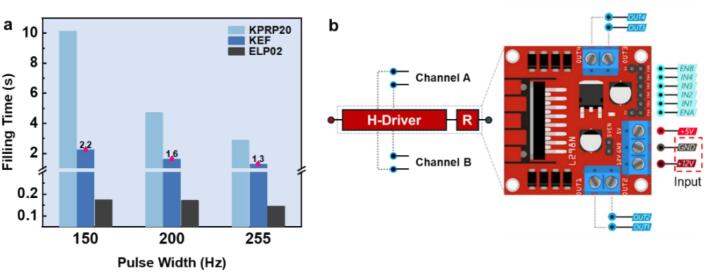
(a) The filling time comparison of 2.0 ml cuvette at specific pulse width values: 150, 200, and 255 Hz. (b) Illustration of the internal resistance on the L298N driver board chip.

To analyze the control characteristics of the pumping system, a detailed comparison among three candidates under typical pulse-width values was conducted. Fig. 15a shows the results from the testing setup, where the filling time of three different DC pumps under typical PWM steps were summarized. It can be observed that the KEF peristaltic pump exhibited a good balance between the actual flowrate and the filling time consumption, indicating mild operating properties for actual application. Specifically, the filling time of a 2.0 mL cuvette can be controlled under 2 s at the normal value of 200 Hz. By conducting this investigation, users can easily compare and screen the optimal external device for actual application based on its multi-thread control functionality. Furthermore, such a synergistic workflow is integrated with the accompanying GUI application ensures an efficient approach to experimentation across different fields.

Compared with the theoretical input voltage of 12.0 V from the DC adapter, the actual maximum voltage measured at the L298N output interface shows a slight drop of 1.50 V. This originates from the internal resistance and switching losses within the H-bridge chip, as shown in Fig. 15b. Consequently, the overall efficiency of the PWM control for the multi-channel pumping system is calculated as 87.6%, with the remaining 12.4% converted into Joule heating. This observation also correlates well with the thermal management and cooling design discussed earlier, confirming the existence of measurable heat generation within the system during prolonged operation. Moreover, these results further demonstrate the advantage of the GUI application, which enables accurate, intuitive, and fully digital control of the pumping parameters without the need for complex manual calibration, enhancing operational efficiency and reproducibility during experimental processes for researchers.

### 7.2 Asynchronous operation test based on the OT-flex

The second test was done on the customized robotic workstation, which was based on the OT-Flex liquid handling robot (running on the Linux system). In this case, the Foehn was integrated with the robotic workstation to accelerate the high-throughput experimentation by enabling the asynchronous operation for actual research application. The integration between the OT-Flex workstation and the Foehn follows a straightforward hardware–software workflow. Specifically, the Foehn system is physically connected to the OT-Flex via the USB serial interface, enabling direct bidirectional communication.

**Fig. 16:**
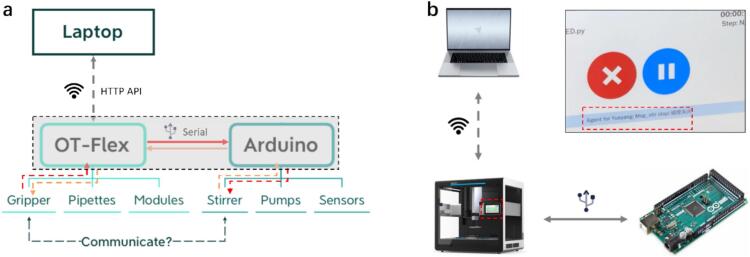
(a) Demonstration of the control architecture between the OT-Flex and the Foehn system, with physical connection marked in the dashed box. (b) Simplified visual illustration of the testing setup, with feedback from the Foehn system displayed on the LED screen of the OT-Flex robot.

The control architecture of the Foehn scheduling hub system is different from the first test done before, as the device of control terminal is the robot instead of the computer. This cascade control architecture allows the robotic workstation to serve as the primary control terminal, while the Foehn system operates as an independent execution node. Therefore, the lab-automation test was realized through the cascade control architecture demonstrated below (Fig. 16a). Since the discussion mainly focused on the test performance of the Foehn system, therefore the mechanism for controlling the customized robotic workstation won’t be included here. In brief, the communication bridge between the OT-Flex and the Foehn relies on the serial port of the physical USB cable, control commands for the Foehn system are embedded within the handshake protocol written in python scripts and uploaded to the OT-Flex through its HTTP API. During execution, the OT-Flex triggers Foehn-related commands asynchronously while continuing its own pipetting and labware-handling tasks. Therefore, the testing setup can be run under a wireless condition as long as the robotic workstation and the laptop stay under the same Wi-Fi environment.

To evaluate the integration compatibility with the robotic workstation, a communication test between the OT-Flex and the magnetic stirrer was carried out. Before the actual integration test, a pre-test for handshake protocol is required to establish the communication chain between those two systems. Herein, a python script was executed to detect the external serial devices based on the Linux system of the robot. After marking down the unique id of the port device, the communication channel can then be constructed via the correct identification inside the python script. Followed by the standard initialization process discussed above, the liquid handling robot successfully established the handshake protocol with the Foehn system via the serial port. This can also be confirmed via the response feedback of the Foehn system after receiving the stop stirring commands from the OT-Flex terminal, with correct response messages displayed on the LED screen of the OT-Flex robot shown in Fig. 16b.

The illustration of the control architecture was summarized for a better understanding of the testing setup, where the testing protocol was first uploaded to the robotic workstation via the HTTP API, followed with an embedded script containing stirring commands for the magnetic stirrer of the Foehn system. By executing the corresponding commands from the robot terminal, the Foehn scheduling hub system can automatically do the asynchronous operation while the OT-Flex is moving labware with the gripper.

**Fig. 17:**
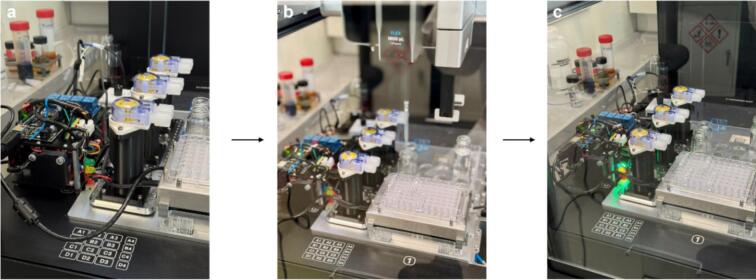
The deployment test of the Foehn system inside the OT-Flex for integration of the asynchronous control functionality in the robotic workstation. (a) Physical connection of the Foehn and the OT-Flex via the USB cable inside the deck. (b) Initialization process of the integrated system. (c) Signal light indication for the normal operation of the pumping system while the OT-Flex carrying labware handling tasks.

To further validate and showcase the asynchronous control process, a complete setup with the embedded Foehn system and external hardware inside the robotic workstation is shown in Fig. 17**a-c**. First, the Foehn system was connected to the workstation through the USB port, ensuring effective physical communication and data synchronization (Fig. 17a). Second, the Foehn system was initialized following the standard integration steps mentioned above, where all onboard LED indicators were activated simultaneously for approximately 3 s, serving as a visual confirmation of successful power-up, firmware loading, and serial communication establishment.

Finally, the testing protocol which contains the asynchronous operation of pumping and stirring commands was successfully sent and executed. The success of the asynchronous and multi-threaded control test was confirmed through both hardware indicators and experimental observation. As shown in Fig. 17**b-c**, the green signal light corresponds to the normal operation of the pumping system, while the yellow light indicates the proper execution of the stirring commands. These LED indicators collectively demonstrate that the real-time communication between the OT-Flex and the Foehn system is both feasible and stable. Notably, the OT-Flex maintained its pipetting and labware-handling operations without any delay or interruption, confirming that data exchange with the Foehn did not interfere with its primary robotic tasks. This observation directly validates the true asynchronous nature of the system—where hardware communication, pumping, and robotic manipulation occur in parallel.

Furthermore, the successful driving of the 96 magnetic stirrer system also shows its strong potential in HTE workflow, since the compact footprint with a compatible system could accelerate and simplify the controlling of mixing process. Meanwhile, the OT-Flex continued its pipetting and labware-moving with its gripper concurrently, demonstrating seamless parallel operation. These results clearly validate the robust asynchronous control capability and multi-threaded performance of the integrated Foehn scheduling hub system. The successful coordination between the Foehn and the robotic workstation highlights its potential as a unified automation platform for complex and parallel experimental workflows.

### 7.3 Conclusions based on the validation tests

The validation and integration studies presented in this work collectively demonstrate that the Foehn scheduling hub provides a reliable and scalable solution for asynchronous operation within automated laboratory workflows. Rather than functioning as a standalone peripheral controller, the Foehn system establishes a coordinated control layer that allows multiple experimental modules to operate concurrently and independently under unified supervision. The results from the first stage confirm that precise, multi-channel pumping and stirring operations can be executed accurately through a fully digital graphical user interface, while maintaining stable hardware–software coordination under varying operational conditions. The consistent voltage stability observed by the L298N drivers and the successful response of the system under variable PWM inputs validated the robustness and accuracy of the hardware-software coordination.

More importantly, the integration test with the OT-Flex robotic workstation further demonstrated the system’s asynchronous and multi-threaded control features. The Foehn system operated in full synchronization with the robotic platform, performing pumping and stirring tasks autonomously while the OT-Flex simultaneously executed pipetting and labware-handling operations. This decoupled execution paradigm overcomes the inherent limitations of sequential workflows and significantly enhances overall efficiency. The successful integration illustrates the strong potential of the Foehn system as a modular control hub for laboratory automation.

Looking beyond the current implementation, the modular and open-source design of the Foehn architecture offers strong potential for scalability and future expansion. The system could be readily deployed across research laboratories in chemistry, biology, and material science, or automated synthesis platforms to streamline repetitive operations and enhance high-throughput setups. From an industrial perspective, the approaches are particularly relevant for automated screening, process development, and material discovery workflow, where equipment utilization, operational flexibility, and scalability are critical. The modular and decoupled control strategy allows peripheral processes to be integrated, replaced, or scaled which is well suited for semi-continuous and high-throughput industrial experimentation. By combining flexible hardware design, customizable software control, and seamless robotic integration, the Foehn scheduling hub represents a meaningful step toward intelligent, distributed, and fully automated experimental ecosystems.

## Ethics statements

This research project mainly focused on the development of the hardware module and its software application in high-throughput experiment procedures; therefore, no human or animal studies were conducted in this work.

## CRediT authorship contribution statement

**Yueyang Gao:** Writing – review & editing, Writing – original draft, Visualization, Validation, Software, Resources, Methodology, Investigation, Formal analysis, Data curation, Conceptualization. **Jacob Danks:** Writing – review & editing, Validation, Investigation. **Simon Dawes:** Writing – review & editing, Validation, Resources. **Maximilian O. Besenhard:** Writing – review & editing, Supervision, Project administration, Funding acquisition.

## Declaration of competing interest

The authors declare that they have no known competing financial interests or personal relationships that could have appeared to influence the work reported in this paper.
